# Royal College of Surgeons of England

**Published:** 1847-02

**Authors:** 


*Royal College of Surgeons of England.*—We cannot afford space for these regulations in full. They may be had at the booksellers, or in our number of last year, of which copies may be obtained at the office. From them it appears that a candidate for the *fellowship* must be twenty-five years of age; that he must have "a competent knowledge" of Greek, Latin, French, and the elements of mathematics. It does not, however, appear that what is called a "competent knowledge" of those matters is defined. He must have been engaged in professional study in schools or hospitals for six years, three of which, at least, must have been passed in *London.* He must have attended a surgical hospital for four years, and a medical one for one, and lectures on anatomy with dissections for three winter sessions; also, lectures on the practice of medicine, and clinical medicine, surgery, and clinical surgery, for two sessions, and on chemistry, materia medica, midwifery, medical jurisprudence, and comparative anatomy, for one session. He must also have served as house-surgeon or dresser in a recognized hospital. Bachelors of arts of "*English*" universities are admitted after five years' study, and are not examined in classics or mathematics. Those who were *members* of the college in September, 1844, are admitted to examination for fellowship when they are of eight years' standing, and those since admitted, or to be admitted members, are admitted to the same examination after twelve years' standing; but the latter must have graduated in an *English* university, or produce a certificate of "competent" classical knowledge. Candidates for the fellowship are examined on "anatomy, physiology, pathology, therapeutics, and surgery," only; and are required to perform dissections and operations on the dead body. It does not appear that they are examined on chemistry, the practice of medicine, materia medica, medical jurisprudence, comparative anatomy, or midwifery. They are, in fact, to be pure surgeons, and not general practitioners, and cannot claim to be appointed to mixed medical institutions, such as the Irish Infirmaries and dispensaries. They are to be what is called in England "pure" surgeons.

Candidates for the *membership* of the College of Surgeons of England are required to produce a certificate of being twenty-nine years of age, and having been engaged "in the acquirement of professional knowledge" for four years, and of having studied practical pharmacy for six months. The meaning of "acquirement of professional knowledge" and "study of practical pharmacy" is not defined. Irish pupils going to London are said to take with them "a certificate" to that effect from any apothecary, which is procured for a trifling douceur. Candidates for the membership are required to attend an hospital for three years, nine months in each year. All the Dublin hospitals are recognized. Candidates are also required to attend lectures on anatomy and physiology, and demonstrations with dissections for three winter sessions, surgery for two, and the practice of physic, chemistry, materia medica, and midwifery, for one. Candidates for the membership are examined in *anatomy and surgery only.* In England the qualification to practice medicine and pharmacy is derived from the Apothecaries' Company, and no man is considered a general practitioner there, or eligible as medical attendant to mixed medical charities, unless he holds their license. The College of Surgeons in England, therefore, *does not examine in practice of medicine or pharmacy, or pretend to give any authority to practice either.* Neither do they require their members to learn or to answer in medical jurisprudence, considering that medical witnesses should have the full qualification of general practitioner. In fact, it appears that the college wish it to be understood that their members are qualified as mere or "pure" surgeons only, and leave them to obtain their qualifications to practice medicine from the medical colleges, and pharmacy at the apothecaries' halls.

With respect to certificates, the London College recognizes all schools and lectures without exception. They state, however, in the above regulations, that, in common with all the other colleges, they reject the certificates of persons who lecture on more than one branch, and therefore refuse the certificates of professors or lecturers who lecture on surgery as well as on anatomy and physiology, but they allow this rule to be evaded. Certificates are not recognized from Dublin, unless the name of the candidate who produces them shall have been returned to the college as having been in attendance on the 25th of November, the 10th of February, and the 10th of May; at which periods the professors, lecturers, and hospital surgeons, are required to return the names of the students then attending, *with the dates of entry*, and periods for which they have entered, "with remarks." "It is required that the dates of commencement and termination be clearly expressed." Students should henceforth be very careful not to rest satisfied with having their names entered into these returns, unless they are really in Dublin and actually "attending" the lectures and hospitals, because any falsehoods in the returns may subject them to the penal provisions of the charter. It is the person who holds, uses, or presents false certificates, who is to be punished, the person who signs and grants them is not held answerable.

There are various methods by which students whose other avocations or limited finances prevent them from complying with the printed regulations of this college can obtain an examination; and as the council appears disposed to afford gentlemen so circumstanced all reasonable facilities towards obtaining a diploma, they can scarcely be blamed for availing themselves of them. An experienced private examiner can explain more to them respecting this matter than we care to mention.—*Dublin Medical Press.*

